# Resistance to neoadjuvant chemotherapy in breast cancers: a metabolic perspective

**DOI:** 10.1186/s13046-025-03500-w

**Published:** 2025-08-11

**Authors:** Manon Desgres, Melis Poyraz, Buse Sari, François P. Duhoux, Cédric van Marcke, Cyril Corbet

**Affiliations:** 1 Pole of Pharmacology and Therapeutics (FATH), Institut de Recherche Expérimentale et Clinique (IREC), UCLouvain, Avenue Hippocrate 57, B1.57.04, Brussels, B-1200 Belgium; 2 Pole of Medical Imaging, Radiotherapy and Oncology (MIRO), Institut de Recherche Expérimentale et Clinique (IREC), UCLouvain, Brussels, Belgium; 3 Department of Medical Oncology, Institut Roi Albert II, Cliniques Universitaires Saint-Luc, Brussels, Belgium; 4 WEL Research Institute, avenue Pasteur 6, Wavre, B-1300 Belgium

**Keywords:** Breast cancer, Neoadjuvant chemotherapy, Metabolism, Tumor microenvironment, Metabolic disorders

## Abstract

Neoadjuvant chemotherapy (NAC) is a cornerstone in the treatment of early-stage high-risk breast cancers (BC), particularly in triple-negative, HER2-positive, and selected hormone receptor-positive subtypes. However, its effectiveness is frequently hindered by intrinsic or acquired resistance, resulting in a significant residual cancer burden (RCB) in more than half of patients. Despite extensive genomic profiling, reliable predictive biomarkers for treatment response remain limited, impeding the development of personalized therapeutic strategies. Emerging evidence highlights tumor metabolic reprogramming as a key non-genetic mechanism contributing to NAC resistance. In this review, we critically examine current advances in metabolic imaging and metabolomics as tools to predict NAC response in BC. We also discuss the role of the tumor microenvironment (TME), including hypoxia and acidosis, in shaping metabolic plasticity and fostering treatment resistance. Furthermore, we explore novel therapeutic strategies targeting metabolic pathways, ranging from enzyme inhibition to dietary interventions, and the use of advanced preclinical models. Together, these insights offer a metabolic framework for overcoming NAC resistance and advancing precision oncology in early-stage BC.

**Supplementary Information:**

The online version contains supplementary material available at 10.1186/s13046-025-03500-w.

## Background

**Fig. 1:**
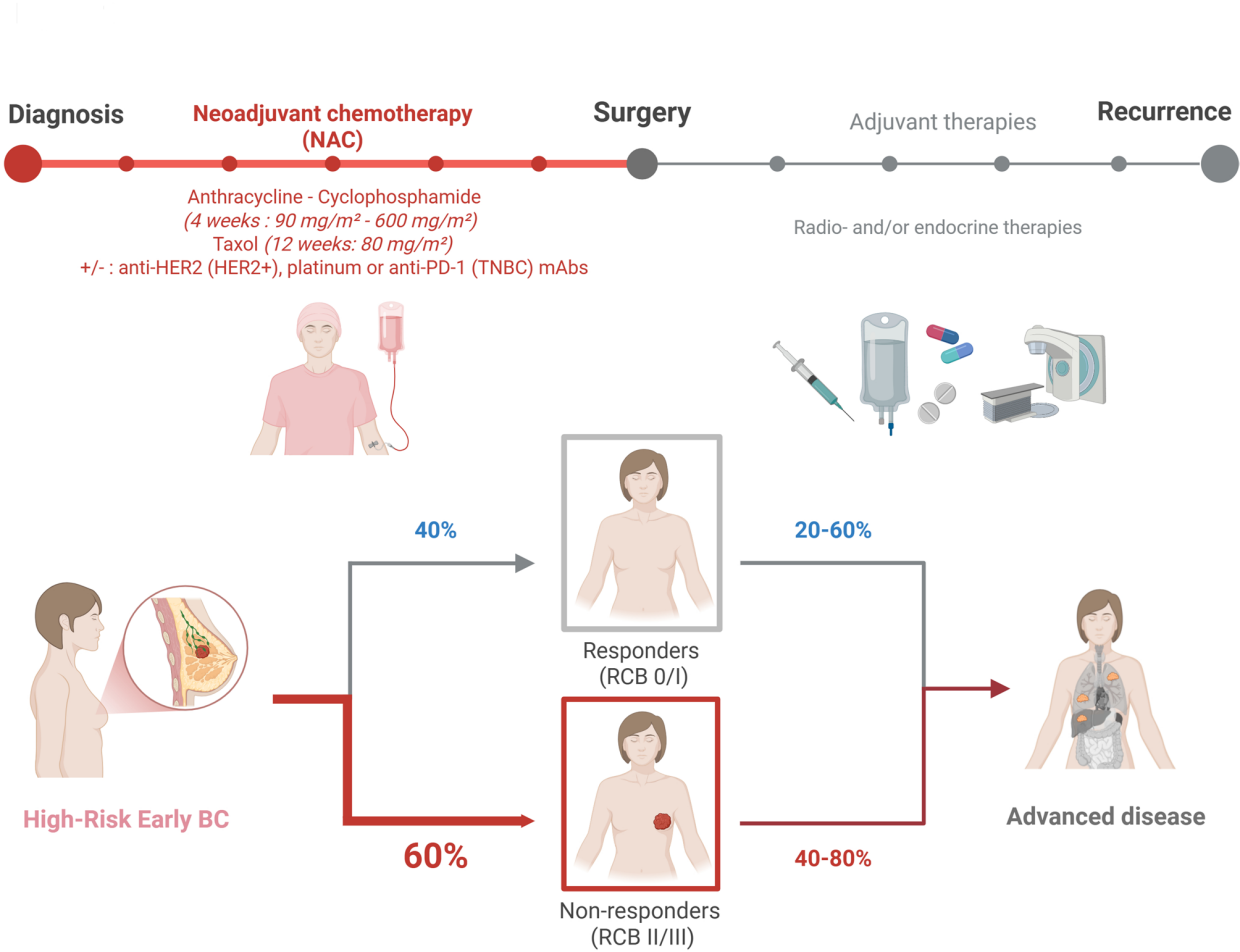
NAC application in clinical management of high-risk early-stage BC. Sequential treatment with anthracyclines/cyclophosphamide and taxanes (supplemented with HER2-targeted therapy or platinum and immunotherapy, in HER2-positive and triple negative BC, respectively) is applied to patients with early-stage high-risk BC NAC, before surgery. Clinical response to NAC is often incomplete, and more than half of BC patients have a residual disease after NAC, as detected by pathological evaluation of the breast and axillary nodes, at the time of surgical resection. Non-responders usually progress with incurable, advanced diseases, despite multimodal adjuvant therapies

Breast cancer (BC) remains the most commonly diagnosed malignancy and the leading cause of cancer-related mortality among women worldwide, despite significant advances in early detection and treatment strategies [1]. Most BC cases are diagnosed at an early, locoregional stage, with *de novo* metastatic disease accounting for approximately 10% of diagnoses [2]. Over the past decade, neoadjuvant chemotherapy (NAC) has become the standard of care for patients with high-risk early-stage BC, mostly triple negative BC (TNBC), HER2-positive tumors but also hormone receptor (HR)-positive BC exhibiting high-risk features such as clinical nodal involvement. Standard NAC regimens typically consist of sequential anthracycline/cyclophosphamide and taxane-based therapies, often in combination with targeted agents such as trastuzumab for HER2-positive tumors or immune checkpoint inhibitors in TNBC [3]. In addition to its systemic benefits, NAC facilitates breast-conserving surgery and provides real-time prognostic information [4]. Achieving a pathological complete response (pCR) following NAC is associated with significantly improved outcomes, especially in aggressive subtypes such as TNBC and HER2-enriched tumors [5, 6]. Nevertheless, the response to NAC remains highly variable. A substantial proportion of patients exhibit residual cancer burden (RCB) at the time of surgery, which is strongly associated with increased risk of recurrence and mortality (Fig. 1) [7]. While TNBC and HR+/HER2- tumors represent opposite ends of the spectrum in terms of pCR rates, both subtypes are linked to high relapse rates when residual disease is present [8–11]. This highlights a critical unmet need: the absence of reliable predictive biomarkers to guide NAC decisions and minimize unnecessary toxicity in non-responders [12]. Although genomic and transcriptomic profiling has provided important insights into tumor heterogeneity and clonal evolution during treatment [13–19], these approaches have yet to yield clinically actionable predictors of NAC response. Increasing evidence points to tumor metabolic reprogramming as a key non-genetic driver of therapy resistance [20–22].

In this review, we examine how metabolic imaging and metabolomics are being employed to predict NAC response and monitor treatment efficacy. We also explore how components of the tumor microenvironment (TME), including hypoxia, acidosis, and stromal interactions, influence metabolic adaptations that promote resistance. Finally, we highlight experimental models and emerging therapeutic strategies aimed at integrating metabolic profiling into clinical decision-making for BC patients undergoing NAC.

## Prediction of NAC response in breast cancer through the lens of cellular metabolism

**Fig. 2:**
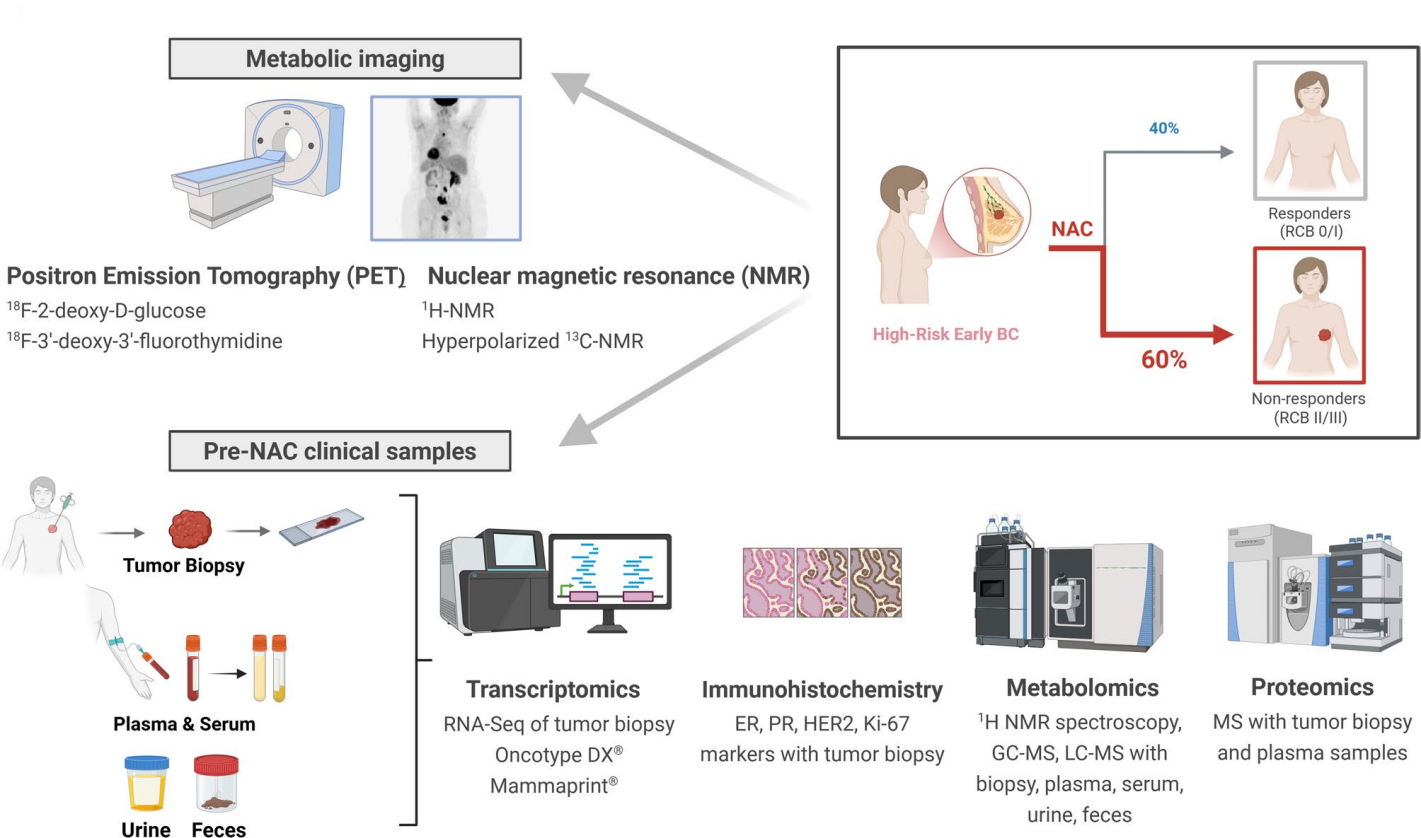
Prediction of NAC response in BC patients through the lens of cellular metabolism. Metabolism-related strategies to predict the final response to NAC include metabolic imaging modalities (PET-scan with ^18^F-tracers, ^1^H-NMR, and hyperpolarized ^13^C-NMR) in treatment-naive BC patients. Transcriptomics, metabolomics, proteomics, and immunohistochemistry analyses can also be used to analyze pre-NAC clinical specimens (e.g. tumor biopsy, serum/plasma, urine, feces)

Predicting response to NAC in early-stage BC remains a major clinical challenge due to the absence of reliable and validated biomarkers. Current decision-making primarily relies on clinicopathological factors such as tumor grade, molecular subtype, tumor-infiltrating lymphocyte density, programmed death-ligand 1 expression, and tumor size, most of which are assessed through immunohistochemistry. However, these markers offer limited predictive accuracy at the individual level [12]. Several studies have explored gene expression profiles from diagnostic biopsies or formalin-fixed paraffin-embedded (FFPE) pre-treatment samples to identify associations with pCR, overall survival (OS), and disease-free survival (DFS) [23–30]. While promising gene signatures have emerged, their clinical utility remains constrained by a lack of validation and standardization. In this section, we examine the potential of metabolism-centered approaches, specifically metabolic imaging and metabolomics, in predicting NAC response, by offering unique insights into the functional state of tumors beyond static genomic profiles (Fig. 2).

### Metabolic imaging and NAC response in breast cancer

Imaging is central to disease staging and treatment monitoring in BC, and metabolic imaging modalities are increasingly investigated for their predictive potential in the neoadjuvant setting. Among these, ^18^F-fluoro-2-deoxy-D-glucose positron emission tomography (^18^FDG-PET) has demonstrated utility in evaluating treatment response, particularly in HR-positive/HER2-negative and triple negative BC [31–41]. The PHERGain phase II trial supported the use of ^18^FDG-PET to guide chemotherapy de-escalation and predict pCR in HER2-positive disease [42]. Early reductions in standardized uptake values (SUV), reflecting tumor glucose uptake, have been correlated with NAC response and long-term prognosis [43], although results across studies have been inconsistent [44]. Notably, combining clinical features with PET/computed tomography (CT)-derived metabolic texture data has improved pCR prediction, particularly in TNBC [45]. However, ^18^FDG-PET reflects only glucose uptake and does not capture downstream metabolic utilization or flux [46]. To address these limitations, alternative metabolic tracers, such as ^18^F-3’-deoxy-3’-fluorothymidine, have been developed as non-invasive tools for early monitoring of treatment response to NAC in patients with locally advanced BC [47, 48]. ^11^C-acetate, ^18^F-fluoroglutamine, and ^18^F-fluoroestradiol, have been used for imaging lipid metabolism, glutamine uptake, and HR activity, respectively [49–52], although their roles in NAC prediction remain largely exploratory.

In parallel, non-radioactive modalities such as ^1^H nuclear magnetic resonance (NMR) spectroscopy have shown promise. Elevated total choline levels detected by ^1^H-NMR have been associated with NAC response in TNBC [53, 54]. More recently, hyperpolarized ^13^C-NMR imaging has enabled real-time monitoring of metabolic flux (e.g. pyruvate-to-lactate conversion), offering dynamic and sensitive assessment of treatment efficacy [55, 56].

Despite these advances, metabolic imaging remains limited by inter-patient variability, the absence of standardized interpretive criteria, and significant intra-tumoral heterogeneity, particularly in luminal tumors [57]. Large-scale, prospective trials are needed to validate its predictive and prognostic utility in clinical practice.

### Metabolomics and NAC response in breast cancer

Metabolomics offers a powerful, systems-level approach to identifying biomarkers of response and resistance to therapy, complementing genomic and proteomic analyses. Various biospecimens, including serum, plasma, tumor tissues, urine, feces, and exosomes, have been analyzed to uncover metabolite signatures predictive of NAC response (Table 1). ^1^H NMR-based serum metabolomics has identified metabolites such as leucine, formate, valine and proline as discriminators of treatment response [58]. Integrated NMR and liquid chromatography-mass spectrometry (LC-MS) approaches have further identified threonine, isoleucine, glutamine, and linolenic acid as predictive markers [59]. In another LC-MS study of 69 BC patients undergoing anthracycline-docetaxel-based NAC, nine metabolites, including prostaglandin C1, oleic acid amide, and vitamin K2, were integrated into a predictive model with excellent diagnostic performance [60].

**Table 1:** Predictive and prognostic values of metabolomics-based data for NAC response in BC patients. Metabolomics analyses on clinical samples (plasma, serum, feces or tumor biopsy), obtained before, during and/or after NAC, from BC patients are indicated in the table. For each study, the analytical method, number of patients, clinical response to NAC, clinical validation level, and major metabolic changes reported, are also described. CE-MS: capillary electrophoresis-mass spectrometry; DFS: disease-free survival; DHA: docosahexaenoic acid; GC-MS: gas chromatography-mass spectrometry; HR MAS MRS: high resolution magic angle spinning magnetic resonance spectroscopy; LC-MS: liquid chromatography-mass spectrometry; NMR: nuclear magnetic resonance; OS: overall survival; pCR: pathological complete response; PR: partial response; RCB: residual cancer burden; SD: stable disease

Timing	Sample type	Analytical method	BC subtype (nb patients)	Clinical response to NAC	Clinical validation level	Metabolic changes	Ref.
Pre-NAC	Plasma	^1^H-NMR	HER2-positive BC (*n* = 43)	pCR (*n* = 24) non-pCR (*n* = 19)	Prospective single arm, phase II monocentric study (NCT02307227)	Identification of valine as part of an immune-metabolomics model to predict NAC response in ER-positive BC patients.	[61]
	Plasma	LC-MS	All BC subtypes (*n* = 75)	RCB0/I (*n* = 16) RCBII/III (*n* = 59)	Prospective monocentric clinical study	Establishment of a predictive model for NAC response based on the differential expression of 19 metabolites.	[64]
	Plasma	UPLC-MS/MS	TNBC (*n* = 88) + healthy controls (*n* = 167)	RCB0/I (*n* = 62) RCBII/III (*n* = 26)	Prospective multicentric clinical study (NCT02276443)	Increased levels of acetylspermidine and diacetylspermine, as well as nine additional metabolites, as predictor of response to NAC.	[62]
	Plasma	UPLC-MS/MS	All BC subtypes (*n* = 99)	pCR (*n* = 54) PR (*n* = 45)	Prospective monocentric clinical study	BC subtype-dependent metabolic alterations between the two pathological response groups. HER2 + BC: amino acids and carbohydrates pathways. TNBC: long-chain FA pathway. Luminal B BC: acylcarnitine pathways.	[63]
	Serum	^1^H-NMR	All BC subtypes (*n* = 80)	RCB0/I (*n* = 16) RCBII/III (*n* = 64)	Prospective monocentric clinical study	Decreased levels of leucine and increased abundance for arginine and formate in non-pCR patients.	[260]
	Serum	^1^H-NMR	TNBC (*n* = 52)	pCR (*n* = 8) PR (*n* = 28) SD (*n* = 16)	Prospective monocentric clinical study	Significant alteration of pathways related to amino acid metabolism as predictor of response to NAC.	[58]
	Serum	^1^H-NMR + LC-MS	All BC subtypes (*n* = 28)	pCR (*n* = 8) PR (*n* = 14) SD (*n* = 6)	Prospective monocentric clinical study	Establishment of a 4-metabolite model (threonine, isoleucine, glutamine, linolenic acid) to predict response to NAC.	[59]
	Serum	GC-MS	All BC subtypes (*n* = 152) + healthy controls (*n* = 155)	Not reported	Prospective multicentric clinical study	Differentially abundant metabolites including 7 for diagnosis, 18 for grading, 23 for staging, and 10 for neoadjuvant status.	[261]
Pre- and during NAC	Plasma	CE-MS + LC-MS	All BC subtypes (*n* = 87)	pCR (*n* = 40) non-pCR (*n* = 47)	Prospective monocentric clinical study	Lower levels of 3-indoxyl sulfate, creatine, and urate in pCR patients, before NAC. Lower levels of 4-methyl-2-oxopentanoate and urate in pCR patients, before the second course.	[65]
	Plasma	GC-MS + LC-MS	HER2-positive BC (*n* = 40)	pCR (*n* = 21) non-pCR (*n* = 19)	Prospective monocentric clinical study	Levels of sophorose, N-(2-acetamido) iminodiacetic acid, taurine and 6-hydroxy-2-aminohexanoic acid to discriminate pCR and non-pCR groups.	[67]
	Feces	^1^H and ^13^C-NMR	HR-positive BC (*n* = 8)	pCR (*n* = 6) non-pCR (*n* = 2)	Prospective monocentric clinical study	Upregulation of amino acids and downregulation of lactate and fumaric acid in patients under the second and third cycles of NAC (vs. pre-treatment). Significant increase of several metabolites (e.g. acetate, creatine, succinate) after NAC in responders, but not in non-responders.	[69]
Pre- and post-NAC	Serum	LC-MS	All BC subtypes (*n* = 35)	PR (*n* = 19) SD (*n* = 16)	Prospective monocentric clinical study	Establishment of a 9-metabolite signature to predict response to NAC.	[60]
	Serum	^1^H-NMR	All BC subtypes (*n* = 80)	RCB0/I (*n* = 16) RCBII/III (*n* = 64)	Prospective monocentric clinical study	Significant association of 4 metabolites (histidine, lactate, serine, and taurine) with DFS + development of a metabolite-related survival score to stratify patients into low- and high-risk relapse groups.	[80]
	Tumor biopsy	HR MAS MRS	All BC subtypes (*n* = 83)	PR (*n* = 51) SD (*n* = 32) Survivors (≥ 5 y) (*n* = 60) Non-survivors (< 5 y) (*n* = 23)	Subcohort from a prospective randomized open-label multicentric clinical study	No difference in the metabolic response between patients with SD and PR. Increased lactate levels in response to NAC in non-survivors. Decreased glycine levels, upon NAC, in survivors.	[83]
Pre-, during and post-NAC	Serum and tumor biopsy	^1^H-NMR	HER2-negative BC (*n* = 118)	RCB0/I (*n* = 34) RCBII/III (*n* = 84) Survivors (≥ 5 y) (*n* = 105) Non-survivors (< 5 y) (*n* = 13)	Prospective phase II multicentric study (NCT00773695)	Higher levels of citrate and lower abundance of phenylalanine, and histidine in RCB II/III vs. RCB 0/I patients. Prediction of 5-year survival from metabolic profiles in tissue, but not serum.	[262]
	Tumor biopsy	HR MAS MRS	HER2-negative BC (*n* = 122)	pCR (*n* = 20) non-pCR (*n* = 102)	Prospective phase II multicentric study (NCT00773695)	Higher glucose and lactate levels in post-NAC samples from responders. Decreased abundance for several metabolites, including creatine, glycine, choline, and succinate in post-NAC samples from responders.	[263]
Post-NAC	Plasma	LC-MS	All BC subtypes (*n* = 92)	pCR (*n* = 54) non-pCR (*n* = 38)	Prospective monocentric clinical study	Higher concentrations of DHA and secondary bile acids in basal and presurgery samples, respectively, in the pCR TNBC patients. Potential of glycohyocholic and glycodeoxycholic acids to classify TNBC patients according to response to NAC and OS.	[81]
	Plasma (exosomes)	LC-MS	All BC subtypes (*n* = 16)	pCR (*n* = 8) non-pCR (*n* = 8)	Prospective monocentric clinical study	Upregulation of succinic acid and L-lactic acid in plasma exosomes from non-responders after NAC.	[68]

Additional studies have confirmed the influence of tumor subtype on metabolomic profiles. For example, valine was identified as a key marker of NAC response in ER-positive tumors [61]. In TNBC, elevated levels of acetylated polyamines (acetylspermidine and diacetylspermine) in non-responders were incorporated into a machine-learning model to enhance predictive accuracy [62]. Subtype-specific plasma metabolite profiles have revealed long-chain fatty acid (FA) enrichment in TNBC and altered amino acid and carbohydrate metabolism in HER2-positive BC [63, 64]. More recently, capillary electrophoresis-MS and LC-MS analyses identified significantly elevated levels of 3-indoxyl sulfate, creatine, and urate in plasma of NAC non-responders [65]. LC-MS profiling of plasma from 30 BC patients further highlighted specific acylcarnitines and amino acids as part of an 18-metabolite predictive signature of treatment response [66]. Metabolomics studies integrating LC-MS and gas chromatography-MS technologies have also investigated biomarkers of response to NAC and HER2-targeted therapies. In HER2-positive BC patients, metabolites such as sophorose, taurine, and N-(2-acetamido) iminodiacetic acid distinguished responders from non-responders. Pathway analysis identified enrichment in glutathione metabolism, protein digestion and absorption, hypoxia-inducible factor 1α (HIF-1α) signaling, and arginine/proline metabolism [67]. Exosome-derived metabolomics further identified succinate and lactate as markers of resistance, along with perturbations in the tricarboxylic acid cycle, glycolysis, gluconeogenesis, porphyrin metabolism, and the urea cycle [68].

Non-blood biospecimens have also shown promise. Fecal metabolomics in a pilot study detected treatment-induced shifts in amino acids, lactate, and fumaric acid [69]. Urinary metabolites such as N1-acetylspermine, spermidine, norepinephrine, and dopamine were higher in responders, offering a non-invasive route for biomarker development [70].

### Proteomics and transcriptomics: identifying metabolism-related biomarkers of NAC response

Proteomic analyses have also yielded insights into metabolism-associated resistance mechanisms. MS-based proteomic profiling of 113 FFPE tumor samples identified proline biosynthesis enzymes (PYCR1 and ALDH18A1) as markers of NAC resistance [71]. Plasma proteomics linked APOC3, MBL2, ENG, and P4HB to pCR and long-term outcomes in NAC-treated patients [72]. In TNBC, treatment resistance was associated with proteins involved in oxidative phosphorylation (OXPHOS), adipogenesis, and FA metabolism [73]. Spatial proteomics of HER2-positive tumors outperformed transcriptomic signatures in predicting pCR [74]. Transcriptomic analyses have likewise identified metabolism-related gene signatures associated with NAC outcomes. Glycolysis-related genes predicted response in TNBC [75], while high expression of lactate dehydrogenase B was linked to chemosensitivity across subtypes [76]. Conversely, OXPHOS gene signatures were associated with resistance and relapse risk [77]. In HER2-positive BC, DUSP4 expression correlated with favorable response to targeted therapy via suppression of glucose-6-phosphate dehydrogenase activity [78].

Collectively, these studies demonstrate that multi-omics analyses, and in particular metabolomics, across a variety of biospecimens and platforms, holds strong potential for guiding patient stratification and personalizing NAC strategies in early-stage BC (Fig. 2). Nevertheless, translation into clinical practice is hindered by variability in sample collection, pre-analytical conditions, tumor heterogeneity, and dietary confounders. Standardization, cross-validation in independent cohorts, and integration into prospective trials are now essential to move these approaches toward clinical utility.

## Prognostic value of metabolic response in BC patients undergoing NAC

### Prognostic significance of metabolic imaging after NAC

^18^FDG-PET has gained recognition as a valuable modality for evaluating metabolic responses to NAC and predicting clinical outcomes. In a systematic review and meta-analysis of 21 studies encompassing 1,630 patients, Han and Choi demonstrated that both interim and post-treatment ^18^FDG-PET assessments were significantly associated with recurrence risk and survival outcomes, including DFS and OS [79]. These findings support the utility of ^18^FDG-PET not only for monitoring treatment efficacy but also for risk stratification and planning adjuvant therapy. Despite this potential, the interpretation of PET data remains complicated by inter-study heterogeneity in imaging protocols, SUV cutoffs, and response definitions. Standardized imaging methodologies and large-scale prospective validation are warranted to fully establish PET-based metabolic response as a prognostic biomarker in routine clinical care.

### Metabolomics and post-NAC prognosis in breast cancer

Metabolomics has emerged as a promising tool to identify prognostic biomarkers that reflect the biochemical landscape of tumors following NAC (Table 1). In a recent study, serum metabolomic profiling before and after NAC in 80 BC patients led to the development of a metabolite-related survival score (MRSS), in which elevated histidine and lactate levels predicted improved DFS, while increased serine and taurine levels were associated with worse outcomes [80]. The MRSS was an independent prognostic factor after adjusting for clinical variables, providing a quantitative approach to post-NAC risk stratification. In TNBC, baseline and pre-surgical plasma metabolomics revealed that higher levels of docosahexaenoic acid and secondary bile acids, including glycohyocholic and glycodeoxycholic acids, were associated with favorable OS and therapeutic response [81]. Fang et al. conducted longitudinal metabolomics analyses in 50 patients with locally advanced BC and identified post-NAC alterations in sphingolipid and amino acid metabolism that correlated with recurrence risk [82]. High-resolution magic angle spinning magnetic resonance spectroscopy of tumor biopsies from 89 BC patients provided further evidence of metabolic alterations correlated with prognosis. Long-term survivors exhibited reduced intratumoral glycine and choline-containing compounds and elevated glucose levels, whereas non-survivors had higher lactate levels, consistent with a more glycolytic and aggressive tumor phenotype [83]. These studies demonstrate the dynamic nature of the metabolome in response to NAC and highlight its potential to inform prognosis, particularly when integrated with clinical and pathological variables.

### Transcriptomics and proteomics: NAC-induced metabolic signatures with prognostic relevance

Beyond metabolomics, transcriptomic and proteomic analyses have uncovered metabolic pathways associated with both NAC sensitivity and long-term outcomes. In a large-scale study involving 634 NAC-treated BC patients, gene sets related to xenobiotic metabolism (e.g. *CYP3A4*, *CYP2A6*) were enriched in non-pCR tumors, while the amino acid transporter *SLC7A5* was differentially expressed between responders and non-responders and was elevated in both primary and metastatic lesions [84]. A separate transcriptomic analysis of matched pre- and post-NAC samples from HER2-negative patients revealed that pathways involving cell proliferation, DNA repair, cellular metabolism, and extracellular matrix (ECM) remodeling were key determinants of recurrence-free survival [85]. Proteomic analyses further support the prognostic value of metabolic regulators. MS-based profiling of tumor tissues identified enzymes involved in proline biosynthesis, namely PYCR1 and ALDH18A1, as markers of tumor relapse and poor OS [71]. In plasma-based studies, elevated levels of MBL2 and P4HB were associated with unfavorable outcomes, including reduced distant metastasis-free survival and OS [72]. These omics-based findings underscore the interplay between metabolic reprogramming and treatment outcomes, providing a molecular basis for refining prognosis after NAC.

## Role of TME-driven metabolic alterations in NAC resistance in BC

The TME is increasingly recognized as a critical regulator of disease progression and treatment resistance in BC patients [86]. Metabolic crosstalk between cancer cells and their surrounding stroma, mediated by oxygen levels, pH, nutrient gradients, and cellular interactions, plays a pivotal role in shaping phenotypic plasticity and therapeutic response [87]. These adaptive responses promote tumor survival under microenvironmental stress and contribute to NAC resistance [88–90]. This section explores how TME-induced metabolic changes influence NAC response and highlights their predictive and therapeutic implications.

### Predictive and prognostic significance of hypoxia in NAC response in BC

**Fig. 3:**
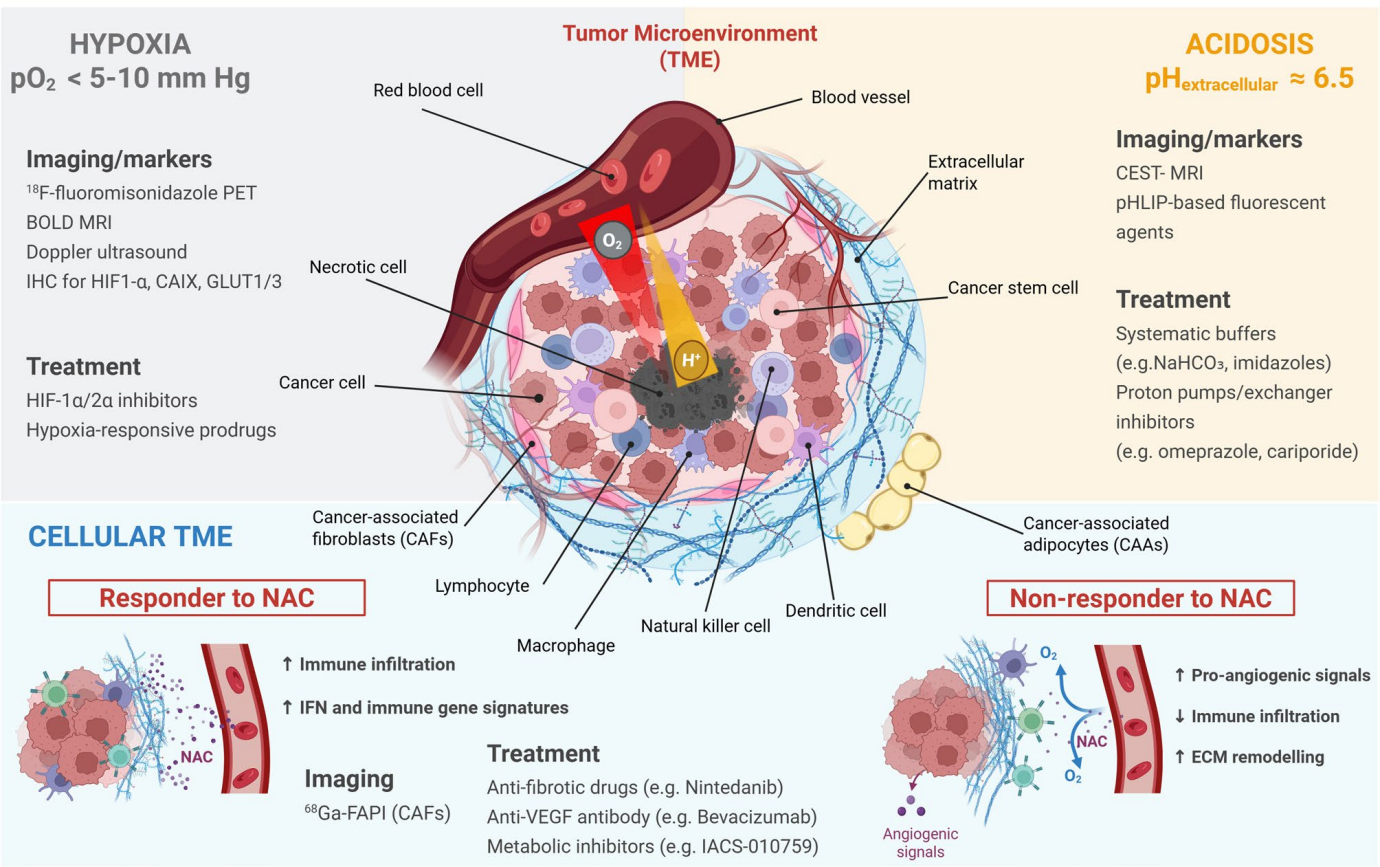
Integrating and exploiting microenvironment-mediated intratumoral metabolic heterogeneity to assess NAC response in BC. Specific conditions within the BC microenvironment, including the existence of hypoxia and acidosis (low pO_2_ and extracellular pH, respectively), and the presence of stromal cells (e.g. cancer-associated fibroblasts) can alter the response to NAC. Imaging modalities and therapeutic strategies have been developed to exploit microenvironmental peculiarities in order to improve NAC response in BC patients

Hypoxia, characterized by inadequate oxygen supply to tissues, is a hallmark of the BC TME and a well-established contributor to tumor progression [91, 92]. Non-invasive imaging modalities such as ^18^F-fluoromisonidazole (^18^F-FMISO) PET and blood oxygen level-dependent (BOLD)-magnetic resonance imaging (MRI) have been used to assess tumor hypoxia pre- and post-NAC (Fig. 3). In a cohort of 33 primary BC patients, higher FMISO tissue-to-blood ratio values correlated with shorter DFS and OS, particularly in ER-positive tumors [93]. Similarly, a window-of-opportunity trial in early HER2-negative BC showed that hypoxic tumors were less likely to achieve pCR after treatment with neoadjuvant nintedanib, a multi-tyrosine kinase inhibitor [94]. Lack of tumor reoxygenation post-treatment was also predictive of residual disease. In a small BOLD-MRI study of seven patients with locally advanced BC, higher oxygen-induced signal changes pre-NAC were associated with subsequent pCR [95]. Other studies have proposed metabolic imaging parameters such as proliferation and perfusion as potential predictors of NAC efficacy [96]. Techniques like ultrasound, diffuse optical spectroscopy imaging, and Doppler ultrasound have shown that early changes in tumor oxygenation may predict treatment response more effectively than size or vascularity changes [97, 98]. However, other imaging methods, such as chemical shift-encoded imaging and lipid quantification, have shown inconsistent predictive value [99].

HIF-1α, a key mediator of hypoxia signaling, has shown variable utility as a biomarker. While some studies linked HIF-1α overexpression to higher pCR rates and tumor grade [100–104], others associated it with poor NAC response and reduced survival [105, 106]. Additional hypoxia markers such as carbonic anhydrase IX and glucose transporters (GLUT1 and GLUT3) have been correlated with reduced relapse-free survival and poor NAC response [107–109]. A validated hypoxia-related gene signature also predicted pCR in HER2-negative BC patients treated with neoadjuvant bevacizumab [110].

Despite promising data, the complexity of hypoxia signaling and TME heterogeneity pose challenges to the clinical utility of hypoxia as a standalone biomarker. Nonetheless, targeting hypoxia-induced resistance using HIF inhibitors [111, 112] or hypoxia-activated prodrugs [113] is a promising therapeutic avenue (Fig. 3).

### Tumor acidosis as s driver of chemoresistance in BC

Acidosis, resulting from aberrant glycolysis and disorganized vasculature, is another key feature of solid tumors including BC. It fosters genetic instability [114, 115], alters gene expression [116], and supports stemness, quiescence, and invasiveness [117, 118].

A major mechanism of acidosis-driven chemoresistance is ion trapping, in which acidic extracellular pH protonates weak base chemotherapeutics (e.g. doxorubicin, mitoxantrone), reducing their membrane permeability and intracellular accumulation [119]. Preclinical studies have shown that buffering agents (e.g. sodium bicarbonate, imidazoles, lysine) [120], proton pump inhibitors (e.g. omeprazole, esomeprazole) [121–123], or proton exchanger inhibitors [124] can partially restore chemosensitivity (Fig. 3).

Acidosis also contributes to resistance through reduced proliferation, upregulation of efflux transporters (ABCG2, P-glycoprotein) [125–129], and induction of the unfolded protein response, particularly via glucose-regulated protein 78, which inhibits apoptosis [130–132]. Metabolically, acidosis promotes autophagy [133, 134], decreases glycolytic flux [135, 136], and enhances FA dependence [137–140]. However, the direct contribution of these metabolic shifts to NAC resistance remains insufficiently defined. For instance, autophagy inhibition via ATG5 silencing failed to reverse doxorubicin resistance in acidic osteosarcoma cells, highlighting tumor-specific and compensatory resistance pathways [123].

Emerging approaches like pH (low) insertion peptides have shown promise in exploiting acidic TME for targeted drug delivery in BC [141–143]. Imaging methods sensitive to acidity, such as MRI-based chemical exchange saturation transfer and optoacoustic techniques, are under investigation for BC detection and staging [144–149], but their predictive value in NAC remains to be established.

### Cancer-associated fibroblasts (CAFs) and immune cells in modulating NAC response

Chemoresistant BC cells frequently shift toward mitochondrial metabolism and utilize alternative substrates such as amino acids and lipids [150, 151]. Residual TNBC lesions post-NAC exhibit elevated metabolic heterogeneity and reliance in non-glucose mitochondrial pathways [152]. Doxorubicin resistance has been linked to GATA3-mediated inhibition of CYB5R2-dependent ferroptosis [153]. However, spatial and temporal resolution of these findings remains limited due to the reliance on in vitro and ex vivo models [154–156]. Intratumoral metabolic heterogeneity, involving dysregulated glycolysis, gluconeogenesis, and FA synthesis, is strongly associated with NAC resistance [157].

Stromal components, including CAFs, adipocytes, and immune cells, facilitate metabolic reprogramming and contribute to therapy resistance. High tumor-to-stroma ratios predict poor outcomes across cancers [158]. Single-cell and spatial transcriptomics have revealed pro-angiogenic signaling and oxygen-dependent metabolic pathways in NAC non-responders [23, 159–165]. CAFs in particular play a multifaceted role in therapy resistance. Highly active fibroblast subtypes associated with fibrosis are linked to poor NAC response and prognosis [166]. Pre-treatment CAF-related gene signatures were predictive of NAC resistance [167, 168], and the anti-fibrotic agent nintedanib improved paclitaxel efficacy and long-term outcomes in HER2-negative BC [169]. Interestingly, SFRP4-positive CAFs were associated with improved survival but paradoxically correlated with reduced NAC response in TNBC [170]. High AU-rich RNA-binding factor 1 expression in CAFs was also linked to poor prognosis in locally advanced BC [171].

CAF-driven resistance mechanisms include secretion of survival-promoting factors [172, 173], induction of stem-like phenotypes [174, 175], and remodeling of the extracellular matrix [168]. In *BRCA1*-mutant TNBC, inflammatory CAF enrichment was linked to angiogenesis and poor response to cisplatin and bevacizumab [176]. NAC has been shown to reprogram fibroblast metabolism toward a glycolytic phenotype, increasing lactate secretion and supporting tumor growth [177]. CD10-expressing CAFs enhance chemoresistance via stearoyl-CoA desaturase-mediated lipid metabolism [178]. Advanced imaging approaches such as PET/CT using ^68^Ga-labeled fibroblast activation protein inhibitor and deep learning-based stroma scoring are being explored to predict NAC response [179–181]. Collectively, these findings emphasize the complexity of stroma-mediated resistance and the value of integrating stromal profiling into NAC decision-making (Fig. 3).

### Cancer-associated adipocytes (CAAs) and obesity in NAC resistance

Adipocytes, the dominant stromal component of breast tissue, interact bidirectionally with cancer cells [182]. CAAs promote chemoresistance by supplying FAs, enhancing lipid oxidation, and promoting stem-like traits [183–185]. Leptin-mediated activation of STAT3 and carnitine palmitoyltransferase 1B supports a chemoresistant phenotype [186]. In obesity, adipose stromal cells adopt pro-tumorigenic phenotypes, myofibroblastic in obese and inflammatory in lean individuals, both contributing to resistance [187].

**Fig. 4:**
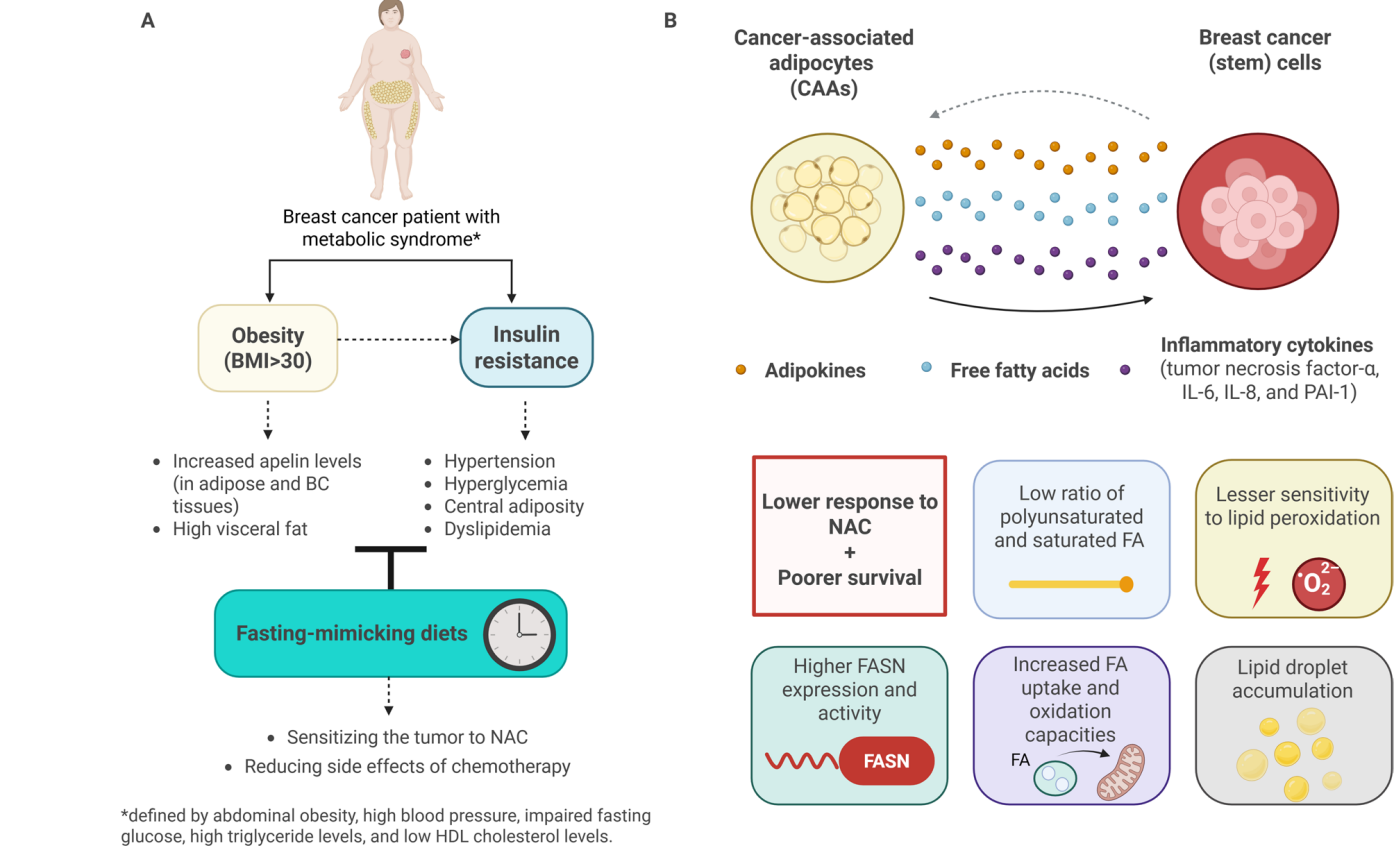
From obesity to the presence of adipocytes within BC microenvironment: influence of lipid metabolism on NAC response. (**A**) Obesity and metabolic syndrome are associated with a lower clinical response to NAC in BC patients. Dietary approaches, such as fasting-mimicking diets, are currently under clinical investigation to assess their influence on NAC efficacy in BC patients. (**B**) Besides the influence of adipokines (e.g. apelin), a lipid-based metabolic communication between cancer-associated adipocytes and BC cells has been reported to support a chemoresistant cancer cell phenotype. NAC-resistant BC cells display major phenotypic changes associated with lipid metabolism, including increased FA uptake, oxidation, and storage capacities. BMI: body mass index; FASN: fatty acid synthase

Epidemiological data supports a strong association between obesity and reduced NAC efficacy [188]. Obesity and elevated apelin levels have been linked to poor response and early recurrence following NAC [189–193]. Visceral adiposity, particularly in postmenopausal patients, correlates with poor NAC outcomes [194–196]. However, adipogenesis-related gene signatures have not reliably predicted NAC response [197]. Metabolic adaptations in resistant cells include altered FA saturation profiles, resistance to lipid peroxidation, increased FA synthase expression, and lipid droplet accumulation [198] (Fig. 4). Despite early promise, trials investigating metformin in NAC settings have produced inconclusive results [199, 200].

Dietary interventions such as fasting-mimicking diets (FMD) have demonstrated NAC-sensitizing effects in preclinical and clinical studies [201, 202]. Phase II trials (DIRECT, BREAKFAST) showed improved outcomes in TNBC and HER2-negative BC patients undergoing NAC with FMD [203–205], and follow-up trials (BREAKFAST-2, DIRECT2) are underway. Metabolic syndrome has also emerged as a negative predictor of NAC response, particularly in TNBC, where it is linked to lower pCR, increased recurrence, and mortality [206, 207].

In summary, CAAs and obesity-driven metabolic changes significantly impact NAC response in BC by reshaping the TME and promoting resistance (Fig. 4). Incorporating host metabolic status into treatment stratification may enhance response prediction and guide personalized therapeutic strategies.

Collectively, findings from metabolic imaging, multi-omics profiling, and TME-focused research underscore the central role of metabolism in determining NAC outcomes in BC. These insights support the integration of metabolic biomarkers into clinical workflows to refine prognosis, monitor treatment response, and inform therapeutic decision-making. Targeting TME-induced metabolic adaptations, either alone or in combination with immunotherapies, offers a promising approach to overcome resistance [208–210]. However, challenges such as intratumoral heterogeneity, interpatient variability, and lack of cross-cohort standardization continue to limit clinical translation. Addressing these barriers remains essential to realizing the full potential of metabolism-informed NAC optimization in early-stage BC.

## Challenges and opportunities for the use of patient-derived BC models to assess NAC response

The development of preclinical models that faithfully replicate the genetic, histological, and functional features of BC is crucial to elucidating intratumoral heterogeneity and the dynamic interplay between tumor cell phenotypes, the TME, and response to NAC. Although widely utilized in high-throughput drug screening, conventional two-dimensional (2D) BC cell line models lack architectural and microenvironmental fidelity and fail to recapitulate the spatial and metabolic heterogeneity of in vivo tumors [211]. These limitations constrain their clinical predictive value and underscore the need for more physiologically relevant platforms. In this context, patient-derived xenograft (PDX) and patient-derived organoid (PDO) models have emerged as complementary tools for modeling therapeutic responses and informing precision oncology strategies.

### Patient-derived xenograft models to assess NAC response

PDX models represent clinically relevant systems for studying BC biology, tumor evolution, and treatment resistance [212, 213]. Successful engraftment is influenced by several factors, including high Ki-67 index, younger patient age, post-NAC status, high histological grade, larger tumor size, and specific morphological features [214]. Engraftment efficiency is particularly elevated in high-grade, ER-negative tumors, suggesting that PDX take rates may reflect aggressive biological behavior and adverse clinical outcomes [215]. In a retrospective analysis of 24 treatment-naïve BC patients followed for a median of 28 months, PDX engraftment correlated with disease relapse [216]. Similar findings were reported in the TOWARDS study, in which PDX take predicted worse survival among patients with newly diagnosed TNBC [217]. Moreover, McAuliffe et al. demonstrated that PDXs were more frequently established from NAC-resistant tumors, supporting their use as prognostic indicators of chemoresistance [218]. However, data from the multicenter BEAUTY trial, which evaluated PDXs generated from pre-NAC biopsy samples of 113 patients, revealed no significant association between engraftment and disease recurrence [219, 220].

PDX models have also provided critical insights into metabolic adaptations following NAC. For instance, Echeverria et al. showed that while residual tumors retained clonal architecture, they exhibited distinct transcriptomic, proteomic, and histological features consistent with a reversible drug-tolerant state characterized by increased OXPHOS activity [221]. Therapeutic targeting of this metabolic shift using the mitochondrial complex I inhibitor IACS-010759 delayed tumor regrowth, highlighting OXPHOS as a potential vulnerability in NAC-resistant TNBC [77]. In another study, lysyl oxidase, a hypoxia-induced ECM-remodeling enzyme, was identified as a mediator of chemoresistance by altering ECM structure and reducing drug penetration [222]. High-throughput drug screening across 16 TNBC PDXs identified the kinesin spindle protein as a promising therapeutic target, while the efficacy of Prima-1Met was associated with glutathione metabolism, suggesting a potential biomarker for response prediction [223].

Despite their advantages, PDX models face notable limitations, including the long latency required for engraftment and expansion, which constrain their real-time clinical utility. Furthermore, the requirement of immunodeficient host mice results in the loss of human stromal and immune components, impeding the study of immune-tumor interactions and limiting translational relevance. These challenges have driven increasing interest in PDO-based systems that offer more rapid, scalable, and immunocompatible platforms.

### Patient-derived organoid models to assess NAC response

PDOs are three-dimensional (3D) cultures derived from patient tumor tissues that retain key histological, genetic, and functional features of the original tumor [224–226]. Compared to 2D cell cultures, PDOs offer a more physiologically relevant model by better preserving tumor architecture and microenvironmental cues, including oxygen gradients [227]. These characteristics making them particularly well-suited for investigating intratumoral metabolic heterogeneity and drug resistance mechanisms [228]. Advances in optical metabolic imaging, through quantification of NAD(P)H and FAD fluorescence lifetimes, have enabled high-resolution visualization of distinct metabolic cell states and improved prediction of therapeutic responses in PDO models [229–231].

The feasibility of integrating matched PDXs and PDOs for drug screening in TNBC has been demonstrated within clinically relevant timeframes [232]. Notably, organoid-forming efficiency from post-NAC residual tumors has been linked to increased recurrence risk and poor clinical outcomes [233]. Additionally, PDOs derived from diagnostic biopsies have shown high predictive accuracy for NAC response [234]. In a recent study, Mazzucchelli et al. established matched pre- and post-NAC PDOs to track tumor evolution and observed enrichment of luminal, treatment-resistant cell populations following chemotherapy [235]. A complementary metabolic profiling study revealed that increased glycolysis and elevated urea cycle enzyme activity were hallmarks of NAC-resistant BC cells, pointing to novel metabolic targets for relapse prevention [236].

Despite their translational relevance, current PDO culture protocols face important lmitations. Standard methodologies typically yield epithelial-only cultures embedded in basement membrane extract and supplemented with niche-specific growth factors, lacking the full cellular diversity of the TME. These conditions often fail to capture the full complexity of the TME, particularly the contribution of non-epithelial cells such as CAFs, immune cells, and vascular components. Moreover, non-tumor cells present in the original tissue, especially fibroblasts, can rapidly outgrow malignant clones unless culture conditions are carefully optimized. The use of inhibitors such as noggin can suppress stromal proliferation and support epithelial expansion but may simultaneously reduce the presence of relevant stromal cell types, limiting the model’s ability to replicate TME-driven resistance mechanisms. To overcome these challenges, co-culture systems incorporating PDOs with additional TME components have been developed [237]. For example, the co-culture of BC organoids with primary macrophages has enabled functional studies of macrophage-induced tumor progression, chemoresistance, and lipid remodeling [238]. Similarly, co-cultures of HR-positive BC PDO with matched CAFs have identified stromal-derived cytokines such as IL-8 and GROα as key mediators of fulvestrant resistance via activation of cytokine-responsive pathways [239]. Metabolically active stromal cells, such as adipocytes, have also been integrated into co-culture models. In one approach, engineered human adipocytes with enhanced glucose and FA consumption suppressed tumor proliferation and metabolic activity through nutrient competition [240]. Although these co-culture systems represent significant progress, the use of primary cells introduces donor-dependent variability, and heterogeneity among tumor subtypes remains a key limitation for model standardization. Moreover, the integration of stromal, immune, and vascular elements into a unified organoid platform remains an ongoing challenge.

Emerging technologies such as microfluidic organ-on-chip and 3D bioprinted platforms offer new opportunities to model dynamic cell-cell and cell-matrix interactions under physiologically relevant settings [241–247]. These systems can simulate perfusion, mechanical forces, and spatial architecture, providing a promising framework for studying chemotherapy resistance under conditions that more closely mimic the in vivo TME.

Despite these advances, few studies have directly addressed immune-mediated mechanisms of NAC resistance in BC. In an immunocompetent 4T1 mouse model enriched for aldehyde dehydrogenase-positive chemoresistant BC stem cells, vaccination with doxorubicin-treated cells elicited robust antitumor immunity and reduced tumor burden and metastasis [248]. In addition, humanized mouse models bearing MDA-MB-231 TNBC tumors with reconstituted immune compartments have demonstrated the efficacy of combined chemo-immunotherapy in controlling tumor growth, highlighting a potential avenue for future clinical application in BC [249, 250].

Collectively, these findings underscore the value of integrating metabolic, immune, and stromal dimensions into patient-derived models to better understand the mechanisms underlying NAC resistance. As technologies continue to evolve, the development of comprehensive, multi-cellular, and metabolically active PDO platforms may significantly advance personalized therapy selection and the discovery of novel therapeutic targets in BC.

## Concluding remarks and perspectives

Resistance to chemotherapy in the neoadjuvant setting remains a major challenge in the clinical management of patients with early-stage, high-risk BC. The current clinical unmet needs are twofold: first, the identification of robust and reliable predictive biomarkers to guide patient selection and treatment stratification, and second, a comprehensive understanding of the molecular mechanisms underlying residual disease following NAC, with the goal of uncovering novel therapeutically actionable targets.

As outlined throughout this review, the integration of metabolomics, metabolic imaging techniques and metabolism-targeted therapies offers a promising strategy to address these challenges [251–253]. These approaches have the potential to improve early prediction of NAC response, uncover novel therapeutic vulnerabilities, and inform treatment adaptation in real time. However, targeting tumor metabolism in this context is complicated by the remarkable plasticity of cancer cell metabolic pathways, which allows for rapid adaptation and contributes to the development of chemoresistance. In addition, many of the most promising metabolic targets lack sufficient tumor specificity, thereby limiting their clinical applicability due to the potential for off-target toxicity and systemic adverse effects. Another important yet often overlooked factor contributing to variability in NAC response is inter-individual pharmacogenomic variability. Polymorphisms in genes encoding drug-metabolizing enzymes such as *CYP1B1*, *CYP2B6*, and *CYP2C9* have been shown to influence NAC efficacy [254–257]. Beyond genetic factors, physiological variables such as the oestrous (menstrual) cycle have recently been implicated in modulating response to chemotherapy in BC patients [258], adding a further layer of complexity to optimizing treatment timing for maximal therapeutic benefit.

This review has also underscored the fragmented nature of current metabolomics research in BC. Many studies are limited by small sample sizes, overfitting, and a lack of robust external validation. Confounding factors such as diet, lifestyle, and environmental exposures can significantly impact metabolic profiles in plasma, tissues, or urine, underscoring the need for standardized protocols in sample collection and analysis. To translate these findings into clinical practice, large-scale, well-controlled studies across diverse populations are urgently needed to validate metabolic biomarkers and establish clinically meaningful thresholds.

Looking ahead, the development of clinically actionable metabolic biomarkers will benefit greatly from the integration of multi-omics technologies with computational modeling. Machine learning and artificial intelligence (AI)-based tools can integrate large-scale, spatially and temporally resolved multi-omics datasets from tumor and associated-TME cells to identify consistent metabolic signatures predictive of NAC response. Although computational algorithms are well established, successful implementation relies on strong multidisciplinary collaboration among clinicians, biologists, and bioinformaticians, as well as enhanced training to advance precision medicine. To maximize reproducibility and clinical impact, future research must also focus on improving the fidelity of disease models such as PDO and PDX, minimizing batch-to-batch variability, and harmonizing biobanking practices. Standardized methodologies for data generation, processing, and integration will be critical to overcoming current barriers and enabling the reliable deployment of metabolic biomarkers in clinical settings [259].

In conclusion, while substantial challenges remain, the convergence of emerging technologies, including advanced metabolic imaging, patient-derived tumor models, and AI-driven analytics, represents a transformative opportunity to refine NAC strategies. By embracing these innovations, the field is poised to shift towards a more personalized, metabolism-informed approach to BC treatment that can improve therapeutic precision, reduce toxicity, and ultimately enhance patient outcomes.

## Supplementary Information

Below is the link to the electronic supplementary material.

Supplementary Material 1

Supplementary Material 2

Supplementary Material 3

Supplementary Material 4

Supplementary Material 5

Supplementary Material 6

## Data Availability

No datasets were generated or analysed during the current study.

## Abbreviations

BC: Breast cancer
CAAs: Cancer-associated adipocytes
CAFs: Cancer-associated fibroblasts
CT: Computed tomography
DFS: Disease-free survival
ECM: Extracellular matrix
ER: Estrogen Receptor
FA: Fatty acids
FFPE: Formalin-fixed paraffin-embedded
FMD: Fasting-mimicking diet
HER2: Human epidermal growth factor receptor 2
HIF: Hypoxia-inducible factor
HR: Hormone receptor
MetS: Metabolic syndrome
MRI: Magnetic resonance imaging
NAC: Neoadjuvant chemotherapy
NMR: Nuclear magnetic resonance
OS: Overall survival
OXPHOS: Oxidative phosphorylation
pCR: pathological complete response
PDO: Patient-derived organoid
PDX: Patient-derived xenograft
PET: Positron Emission Tomography
RCB: Residual cancer burden
SUV: Standardized uptake values
TME: Tumor microenvironment
TNBC: Triple-negative breast cancer
UPR: Unfolded protein response
