# Supplementary figures and images for: Resistance to neoadjuvant chemotherapy in breast cancers: a metabolic perspective
Source: J Exp Clin Cancer Res. 2025 Aug 11;44:234. doi: 10.1186/s13046-025-03500-w (PMC12337530; doi:10.1186/s13046-025-03500-w)

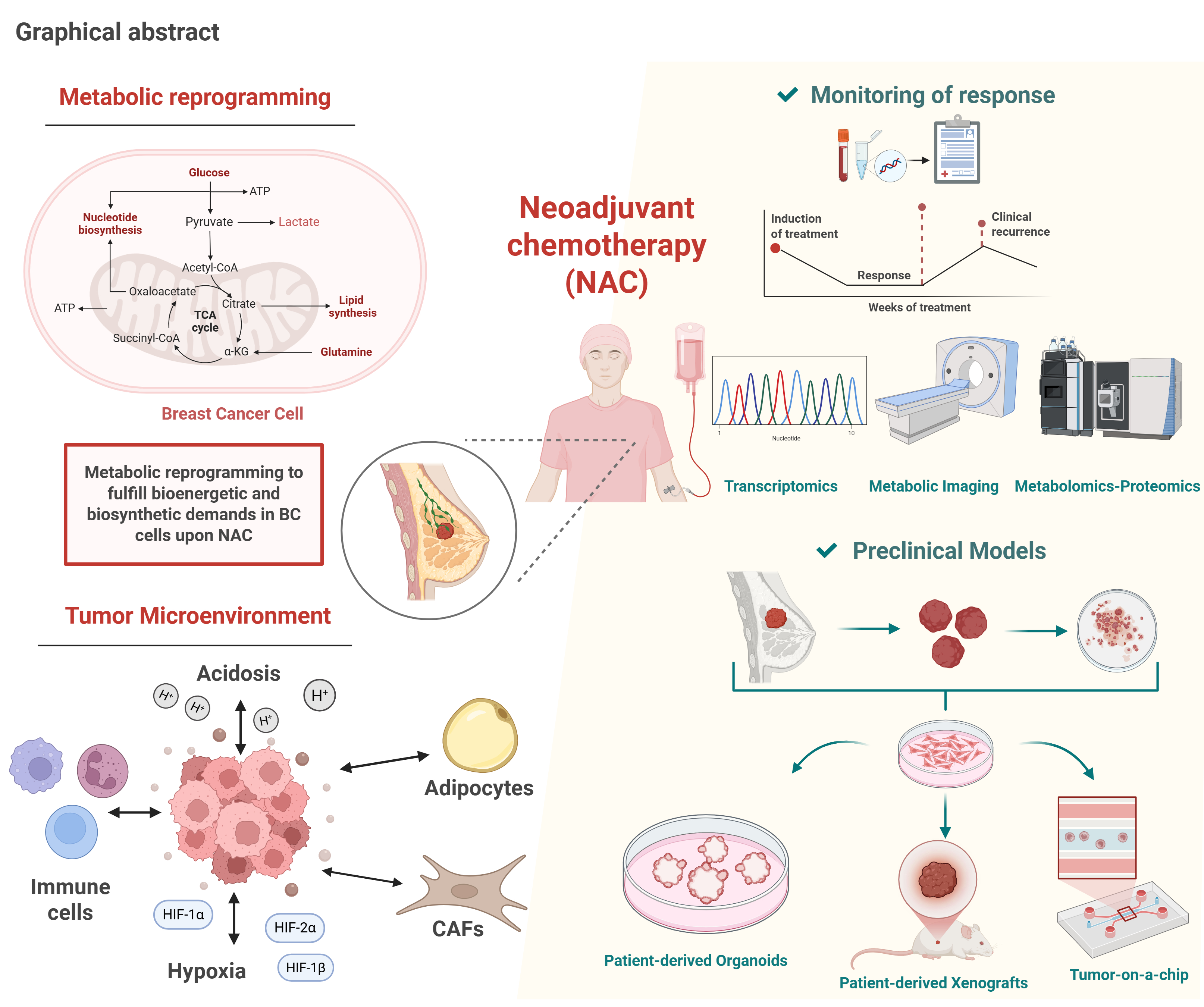

Supplement: Supplementary file 1 — Supplementary Material 1 [file 13046_2025_3500_MOESM1_ESM.jpeg]
